# Viability Status-Dependent Effect of *Bifidobacterium longum* ssp*. longum* CCM 7952 on Prevention of Allergic Inflammation in Mouse Model

**DOI:** 10.3389/fimmu.2021.707728

**Published:** 2021-07-20

**Authors:** Marcelina Joanna Pyclik, Dagmar Srutkova, Agnieszka Razim, Petra Hermanova, Tereza Svabova, Katarzyna Pacyga, Martin Schwarzer, Sabina Górska

**Affiliations:** ^1^ Laboratory of Microbiome Immunobiology, Hirszfeld Institute of Immunology and Experimental Therapy, Polish Academy of Sciences, Wroclaw, Poland; ^2^ Laboratory of Gnotobiology, Institute of Microbiology, Czech Academy of Sciences, Novy Hradek, Czechia

**Keywords:** probiotic, heat inactivation, intranasal administration, *Bifidobacterium*, Ovalbumin sensitization, allergy

## Abstract

The classical definition of probiotics states that bacteria must be alive to be beneficial for human organism. However, recent reports show that inactivated bacteria or their effector molecules can also possess such properties. In this study, we investigated the physical and immunomodulatory properties of four *Bifidobacterium* strains in the heat-treated (HT) and untreated (UN) forms. We showed that temperature treatment of bacteria changes their size and charge, which affects their interaction with epithelial and immune cells. Based on the *in vitro* assays, we observed that all tested strains reduced the level of OVA-induced IL-4, IL-5, and IL-13 in the spleen culture of OVA-sensitized mice. We selected *Bifidobacterium longum* ssp. *longum* CCM 7952 (Bl 7952) for further analysis. *In vivo* experiments confirmed that untreated Bl 7952 exhibited allergy-reducing properties when administered intranasally to OVA-sensitized mice, which manifested in significant suppression of airway inflammation. Untreated Bl 7952 decreased local and systemic levels of Th2 related cytokines, OVA-specific IgE antibodies and simultaneously inhibited airway eosinophilia. In contrast, heat-treated Bl 7952 was only able to reduce IL-4 levels in the lungs and eosinophils in bronchoalveolar lavage, but increased neutrophil and macrophage numbers. We demonstrated that the viability status of Bl 7952 is a prerequisite for the beneficial effects of bacteria, and that heat treatment reduces but does not completely abolish these properties. Further research on bacterial effector molecules to elucidate the beneficial effects of probiotics in the prevention of allergic diseases is warranted.

## Introduction

Food and respiratory allergies have become a significant health burden in both developed and developing countries. Recent data indicate that the percentage of people demonstrating symptoms of allergy reaches up to 40% of the world population (1, 2). Allergies are characterized by an abnormal reaction of the body to a commonly harmless substance. Hypersensitivity type I reaction is associated with Th2 polarized lymphocyte response and involves production of IL-4, IL-5, and IL-13 cytokines and allergen-specific IgE antibodies. In particular, IL-4 and IL-13 regulate many aspects of allergic response, *i.e.*, in B cells prompt the immunoglobulin switch to IgG1 and IgE, activate and recruit eosinophils and induce mucus hypersecretion in lung tissue (3). In respiratory allergies, nasal and lung epithelial cells are the first cells encountering allergen, such as house-dust mite faeces, fungal spores or plant pollen. Besides being a physical barrier separating the internal milieu from the external environment, epithelial cells also play an important role in initial recognition of antigens and regulation of the immune responses to them (4). Moreover, epithelial cells are able to secrete a large number of cytokines and chemokines that regulate the activation of T and/or B lymphocytes, eosinophils, mast cells, and ILC2 cells (5, 6).

Growing evidence suggests that probiotics could be a promising strategy in the prevention/treatment of allergy diseases (7). Recently, several studies have shown that prophylactic administration of probiotics attenuates the production of pro-inflammatory responses in house dust mite-induced asthma model (8, 9). Along with these results, it has been shown that intranasal administration could be more effective compared to standard intragastric route (10). It has been also noticed, that probiotics interaction with mucosa of the respiratory tract leads to the activation of both local and systemic immune responses (11, 12).

While probiotics are generally regarded as safe, the administration of live bacteria to certain groups of patients could cause bacteremia (13). This especially concerns people with weakened immune system *e.g.*, new-borns or elderly people (14, 15). Until recently, it was considered that probiotics should be administered alive in order to exert their beneficial properties (16, 17). However, there is an accumulating evidence showing that even administration of inactivated bacterial strains and bacteria-derived molecules is able to ameliorate the development of airway allergy (18–21). For example, it has been shown that administration of thermally inactivated *Enterococcus feacalis* reduced nasal mucosa swelling and decreased eosinophil level in a mouse model of allergic rhinitis (22). Similarly, thermally inactivated *Lactobacillus* (*L.*) *casei* Shirota inhibits the production of IgE in mouse model of allergy, which may indicate a protective role in allergy modulation (23). Even so, depending on the bacterial species or bacterial strain, desired probiotic properties may be retained only partially or lost completely during heat inactivation (24).

In this study we characterized the viability status-dependent physical and immunomodulatory properties of four strains belonging to different *Bifidobacterium* species. On the basis of the potential to downregulate the allergic and inflammatory cytokine response, we selected *Bifidobacterium longum* ssp. *longum* CCM 7952 (Bl 7952) strain to further investigate the impact of thermal inactivation on prevention and modulation of allergic immune response to ovalbumin (OVA) in a mouse model of allergy. We found that intranasal administration of untreated Bl 7952 strain prevented the development of allergic lung inflammation and modulated both local and systemic OVA-specific immune responses. These immunomodulatory properties were partially lost when heat-treated Bl 7952 was used.

## Materials and Methods

### Cultivation and Inactivation of Bacterial Strains

Four *Bifidobacterium* strains: *B. longum* ssp. *longum* CCM 7952 (Bl 7952), *B. longum* ssp*. infantis* CCDM 369 (Bin 369), *B. animalis* CCDM 218 (Ban 218), and *B. adolescentis* CCDM 373 (Bad 373) were obtained from the Collection of Dairy Microorganisms (Laktoflora, Milcom, Tábor, Czech Republic). They were isolated from fecal samples of healthy adults or breast-fed infants. Stocks of strains were kept at −80°C in MRS (De Man, Rogosa and Sharpe medium, Sigma Aldrich, USA) with 0.05% L-cysteine (Sigma Aldrich, USA) and 20% glycerol. The isolates were cultivated for 48 or 72 h in MRS broth (Sigma Aldrich, USA) with 0.05% L-cysteine (Merck Millipore, Massachusetts, USA) at 37°C in anaerobic conditions (80% N_2_, 10% CO_2_, 10% H_2_). They were centrifuged (4,500 × g, 15 min, 4°C) and washed with sterile phosphate-buffered saline (PBS). The number of cells was determined by CFU counting on MRS agar plates with 0.05% L-cysteine after 48 h of anaerobic incubation or by QuantomTx Microbial Cell Counter (Logos Biosystems, South Korea) and associated with the values obtained during the measurement of OD_600_. Bacterial survival in PBS (HIIET PAS, Poland) after 72 h at 4°C was checked by plate culture and CFU counting. Heat inactivation was performed at 65°C for 1 h, and samples were stored at 4°C until use. Loss of viability was examined by culture on MRS agar plates supplemented with L-cysteine in anaerobic conditions.

### Scanning Electron Microscopy at Low Voltage

The untreated bacteria (10^7^ CFU/ml) were plated onto an MRS Agar plate and after 48 h of incubation were pressed against a silicon chip (7 × 7 mm), while the heat-treated bacteria were prepared in a volume of 1 ml in an Eppendorf in which a silicon chip was placed. In both cases, a 2 min incubation was performed, and then the chip was removed for further preparation steps for imaging. The bacteria-containing chip was rinsed with PBS (HIIET PAS, Poland) at room temperature and immersed in 2.5% glutaraldehyde (Sigma Aldrich) in 0.1 M cacodylate buffer. Fixation was continued for 30 min, followed by washing (5 × 30 min, 4°C) with 0.1 M cacodylate buffer and dehydration in serial concentrations of ice-cold methanol (25, 40, 60, 80, and 100%). All samples designed for imaging at room temperature underwent critical point drying with 100% methanol exchanged for liquid CO_2_ in an automated manner (CPD300 AUTO, Leica Microsystems, Germany) and were imaged under a cross-beam scanning electron microscope equipped with a Schottky field-emission cathode (Auriga 60, Carl Zeiss) at 1.2 kV accelerating voltage.

### Physical Measurements of Untreated and Heat-Treated Bifidobacteria

Two samples of each *Bifidobacterium* strain at a density of 10^7^ CFU/ml were prepared in 300 μl of PBS. Inactivation was performed by heat treatment at 65°C for 1 h. After cooling to room temperature (25°C), both untreated (UN) and heat-treated (HT) were transferred to the measuring cuvette and analyzed in terms of their size (Dynamic Light Scattering, DLS) and zeta potential (Electrophoretic Light Scattering, ELS) using Zetasizer Nano ZS (Malvern Panalytical, UK). Measurements were made in milli-Q water in a Folded Capillary Cell (DTS1070, Malvern Panalytical). Each probe was measured five times at 25°C (at least 15 runs, voltage adjusted automatically by the software). To perform calculation dispersant RI 1.333 and viscosity (Cp) as 0.87 were used.

### Cell Lines and Culture Conditions

A cancerous epithelial line TC-1 (ATCC CRL-2785) isolated from murine lungs immortalized with human papillomavirus 16 (HPV-16) E6/E7 and c-Ha-Ras cotransformed was maintained in Dulbecco’s Modified Eagle Medium (DMEM, Gibco, Thermo Fisher Scientific, USA) with 10% fetal bovine serum (FBS, Gibco, Thermo Fisher Scientific), 100 U/ml penicillin, 100 µg/ml streptomycin and 2 mM L-glutamine (Gibco).

JAWS II cell line (ATCC CRL-11904) was established from bone marrow-derived dendritic cells of C57BL/6 mouse and was maintained in Alpha Minimum Essential Medium Eagle (Alpha MEM, Gibco) with 10% FBS, 100 U/ml penicillin, 100 µg/ml streptomycin, 2 mM L-glutamine, 1 mM sodium pyruvate (Sigma Aldrich), and 5 ng/ml granulocyte-macrophage colony-stimulating factor (GM-CSF, Invitrogen, Thermo Fisher Scientific).

Both cell lines were cultivated in an incubator at 37°C, 5% CO_2_. Culture media were changed every two to three days, and the cell lines were trypsinized with 0.25% trypsin-EDTA pH 7.2 solution when 80% confluency was reached.

### *Bifidobacterium* Engulfment by Epithelial Cells

TC-1 cells were seeded at density of 0.4 × 10^6^ cells/ml on 24-well plate in medium without antibiotics (DMEM, 10% FBS) and left for 5 h to adhere at 37°C, 5% CO_2_. Next, UN and HT bifidobacteria were stained as in the manufacturer protocol with SYTO™ 9 (Thermo Fisher Scientific) and were added to the epithelial cells in the ratio of 1:10 (TC-1:bacteria) in antibiotic-free medium. Bacteria were counted with QuantomTx Microbial Cell Counter (Labos Biosystem). Samples were incubated overnight and the next day, TC-1 cells were washed twice with pre-warmed PBS. Cells were detached from the plate with trypsin and analyzed by the BD FACSCalibur Flow Cytometry System (BD Biosciences, USA) in terms of green fluorescence. TC-1 cells mixed with PBS were used as negative control. Each experiment was repeated three times.

### *Bifidobacterium* Transfer Between Epithelial and Dendritic Cells

TC-1 cells were grown and loaded with stained bacteria as described above. Simultaneously, JAWS II cells were cultivated and stained with red fluorescent dye PKH26 (Sigma Aldrich). Next, they were added to the TC-1 cells in an approximate ratio of 1:1 in JAWS II complete medium. Cells were co-cultured for 4 h in the incubator (37°C, 5% CO_2_). Cells were detached from the plate with trypsin and analyzed by the BD FACSCalibur Flow Cytometry System in terms of green and red fluorescence. Each experiment was repeated three times.

### TC-1 Cell Stimulation With Bifidobacteria

TC-1 cells were seeded at density of 2 × 10^6^ cells/ml on 96-well plate in medium without antibiotics (DMEM, 10% FBS) and stimulated with UN or HT bifidobacteria strains in the ratio of 1:10 (TC-1:bacteria). TC-1 cells stimulated with PBS were used as controls. The supernatants were collected after 20 h of incubation at 37°C, 5% CO_2_. The levels of IL-6 and MCP-1 were quantified using enzyme-linked immunosorbent assay (ELISA) detection kits (Invitrogen or BD OptEIA Pharmingen, USA) according to the instructions of the manufacturer. Each experiment was repeated three times.

### Bone Marrow-Derived Dendritic Cell Isolation and Stimulation

The mouse bone marrow precursors were isolated from femurs and tibias of BALB/c mice (8-weeks old). Cells were cultured at density of 4 × 10^6^ cells/ml on Petri dishes in 10 ml of Roswell Park Memorial Institute (RPMI 1640, Sigma Aldrich) culture medium containing 10% FBS, 150 μg/ml gentamycin (Sigma Aldrich) and 20 ng/ml GM-CSF (Invitrogen). Fresh medium was added at days 3 and 6, and BMDCs were used for experiments on day 7 of culture. BMDCs (0.5 × 10^6^ cells/ml) were stimulated with UN or HT bacteria in ratio of 1:10 (BMDCs:bacteria) for 20 h. Control BMDCs were stimulated with PBS. Levels of IL-10 and IL-12p70 in culture supernatants were determined by ELISA Ready-Set-Go! kits (eBioscience, Germany, San Diego, CA, USA) according to the instructions of the manufacturer. Each experiment was repeated twice.

### Stimulation of TLR2, TLR4, NOD2, and NOD1 Receptors

The human embryonic kidney cell line HEK293 stably transfected with a plasmid carrying the human (h) TLR2/CD14 gene was kindly provided by M. Yazdanbakhsh (Leiden, The Netherlands), and hTLR4/MD2/CD14 by B. Bohle (Vienna, Austria). Cells transfected with hNOD2 and hNOD1 were purchased from InvivoGen (InvivoGen, USA). The cells were stimulated for 20 h with UN and HT bifidobacteria at concentrations of approximately 1 × 10^7^ CFU/ml in 96-well plates. TLR2 ligand Pam3CSK4 (1 µg/ml, InvivoGen), NOD2 ligand muramyl dipeptide (MDP; 100 ng/ml; InvivoGen), TLR4 ligand ultrapure LPS-EB (1 µg/ml, Sigma Aldrich), and NOD1 ligand acylated derivative of the iE-DAP dipeptide (C12-iE-DAP; 100 ng/ml; InvivoGen) were used as positive controls. Culture supernatants were harvested and human IL-8 concentrations, as level of respective receptor activation, were analyzed by ELISA (Invitrogen, Thermo Fisher Scientific) according to the instructions of the manufacturer. Each experiment was repeated three times.

### Stimulation of Splenocytes From OVA-Sensitized Mice With UN and HT *Bifidobacterium* Strains

Immunomodulatory potential of *Bifidobacterium* strains was tested *ex vivo* on splenocytes derived from ovalbumin (OVA)-sensitized BALB/c mice (8–12 weeks of age; n = 3) in four independent experiments. Female mice were sensitized by two intraperitoneal (i.p.) injections of 10 µg of OVA (Sigma Aldrich, grade V) mixed with 0.65 mg/100 μl of Alum (Serva, Germany) in final volume of 200 μl in a two-week interval. Seven days after the second immunization, mice were anesthetized by 3% isoflurane and euthanized by cervical dislocation. Spleens were aseptically removed and prepared by disruption of the tissues through a 70 µm cell strainer into culture medium RPMI 1640 with 10% FBS, 100 U/ml of penicillin, 100 µg/ml streptomycin, and 10 mM HEPES. Splenocytes (5 × 10^6^ cells/ml) were re-stimulated in 96-well flat-bottom plates with OVA (500 μg/ml, Worthington, USA) together with UN/HT bifidobacteria in a ratio of 1:10 (cells:bacteria) for 72 h. Splenocytes stimulated only with OVA were used as baseline of cytokine response to OVA re-stimulation. Unstimulated splenocytes were used as a negative control. Concentration of cytokines was measured in supernatants by the Milliplex Map Mouse Cytokine/Chemokine Panel (IL-4, IL-5, IL-13, and IFN-*γ*) according to the instructions of the manufacturer and analyzed with Luminex 2000 System (Bio-Rad Laboratories, CA, USA).

### *In Vivo* Effect of UN and HT *B*. *longum* ssp. *longum* CCM 7952 (Bl 7952) in Mouse Model of OVA-Induced Allergy

#### Animals

Pathogen-free female BALB/c mice (6–8 weeks of age) were maintained in individually ventilated cages (IVC, Tecniplast, Italy) with a 12:12-h light–dark cycle and free access to water and sterile irradiated diet (Altromin Spezialfutter GmbH & Co. KG, Lage, Germany). The animal experiments were approved by the committee for the protection and use of experimental animals of the Institute of Microbiology, The Czech Academy of Sciences (no. 91/2019).

#### Experimental Design

**Figure 1:**
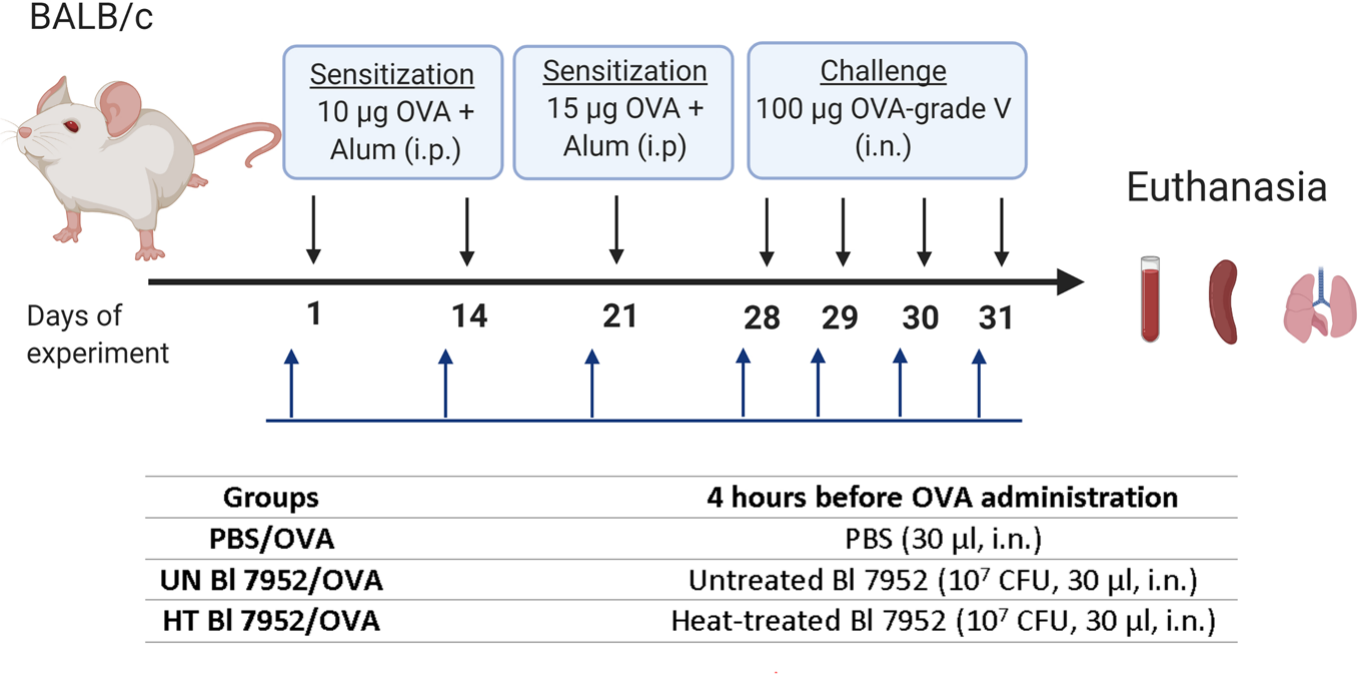
Experimental design—bacterial administration and systemic sensitization and challenge of BALB/c mice to OVA. Female BALB/c mice 6–8-week-old (n = 6 to 8) were i.p. sensitized with 10 µg of OVA + Alum on days 1 and 14 of experiment, boosting dose of 15 µg of OVA + Alum was administrated on day 21. Seven days after third immunization, the challenge with 100 µg of OVA was performed for 4 consecutive days. Untreated (UN Bl 7952) or heat-treated (HT Bl 7952) bifidobacteria in dose 1 × 10^7^ CFU/30 µl or PBS were administered intranasally 4 h before each sensitization and challenge. Mice were euthanized on day 32, and samples were collected (Created with BioRender.com).

Mice (*n* = 6–8 per group) were divided into three experimental groups: PBS/OVA, untreated (UN) Bl 7952/OVA or heat-treated (HT) Bl 7952/OVA. Mice were sensitized by i.p. injections of 10 µg of OVA (Sigma Aldrich, grade V) mixed with PBS and adsorbed on 0.65 mg of Alum (Serva) in a final volume of 200 μl, on days 1 and 14. Third boosting immunization (i.p.) was performed one week after the second injection by 15 µg OVA (Sigma Aldrich, grade V) in PBS mixed with Alum (0.65 mg per dose) (200 µl). Seven days after the third immunization, mice were anesthetized by 3% isoflurane and challenged by intranasal administration of 100 µg of OVA in PBS in a dose of 30 µl. The procedure was repeated each day for 4 consecutive days. Four hours before each sensitization and OVA-challenge application, mice were anesthetized by 3% isoflurane and PBS; untreated or heat-treated Bl 7952 (1 × 10^7^ CFU/30 µl) were applied intranasally (i.n.). Mice were anesthetized by 3% isoflurane and euthanized on the day following the fourth challenge and samples [serum, spleen, bronchoalveolar lavage (BAL), lung lobes] were collected (**Figure 1**). The experiment was repeated twice yielding similar results; data from a representative experiment are shown.

#### Quantification of Cytokine Production by Splenocytes

Spleen cell suspensions (5 × 10^6^ cells/ml) were cultured on 96‐well flat‐bottom plates in 200 µl of complete RPMI 1640. Cells were re-stimulated with OVA (500 µg/ml; Worthington, USA) or left unstimulated and cultured at 37°C, 5% CO_2_ for 72 h. Supernatants were collected and stored at −40°C until further analysis. The levels of IL‐4, IL‐5, IL‐10, and IL‐13 were determined using a Mouse Cytokine/Chemokine Multiplex Immunoassay (Millipore) and analyzed with a Luminex 200 System (Bio‐Rad Laboratories). IFN-*γ* was assessed by ELISA kit (R&D Duo set System, USA) according to the instructions of the manufacturer.

#### Histopathological Evaluation of Lung Inflammation

Small lung lobes were fixed in 4% paraformaldehyde for 24 h and stored in 80% ethanol. After embedding in paraffin, 5 µm-thick sections were treated with periodic acid-Schiff (PAS) staining. Histological pathology scoring was evaluated using light microscopy (×100 magnification) according to Drinić et al. (25) and expressed as: (i) perivascular and peribronchiolar inflammation (grade: 0  =  no changes; 1 = few perivascular and peribronchiolar inflammatory cells; 2 = moderate numbers of cell infiltrations on several perivascular and peribronchiolar sites; 3 = large number of diffuse infiltrated cell cuffs), (ii) the presence of leukocytes in alveolar spaces (grade: 0 = no cells; 1 = 2–4 cells; 3 = 4–10 cells; 3 = more than 10 cells), and (iii) number of PAS positive cells per 50 counted bronchoalveolar epithelial cells (0 = no cells; 1 = <12; 2 =12–25; 3 = >25). The histopathological score is expressed as a sum of single scores divided by 3.

#### Analysis of Bronchoalveolar Lavage

BAL fluid (BALF) was collected from each animal *via* cannulation of the exposed trachea and gentle flushing of the lungs with 2 × 0.5 ml of sterile Dulbecco’s Phosphate Buffered Saline (D-PBS, Gibco). BALF was centrifuged (400 × g, 7 min, 4°C), and the supernatant was collected and stored at −40°C. Cell pellet was recovered for cellular analysis by 200 µl of RPMI medium. After cell counting, cells were spun down onto microscope slide, fixed with methanol and stained by Dip-Quick-stain kit according to instruction of the manufacturer (Medical products, Czech Republic). Cytospin preparations were differentiated according to standard morphologic criteria by counting 200 cells *via* light microscopy (26). The levels of IL-4, IL-5, IL-10, and IFN-*γ* were determined using a Mouse Cytokine/Chemokine Multiplex Immunoassay (Millipore) or ELISA kit (R&D Duoset System, Minneapolis, MN, USA) according to the instructions of the manufacturer.

#### Humoral Immune Response in Serum and BALF

Levels of OVA-specific IgG1, IgG2a, and IgA in serum or BALF were determined by ELISA as previously described (27). Briefly, the 96-well microtiter plates (Nunc MaxiSorp, Thermo Fisher Scientific) were coated with OVA (5 μg/ml), and mouse serum samples were diluted 1:10,000 for IgG1, 1:100 for IgG2a, and 1:10 for IgA quantification. Rat anti-mouse IgG1, IgG2a and IgA antibodies (1:500; Pharmingen, BD Biosciences, USA) were applied, followed by peroxidase-conjugated mouse anti-rat IgG antibodies (1:2,000; Jackson Immuno Labs, USA). Antibody levels were reported as optical density (OD) at wavelength λ = 405 nm.

The activity of OVA-specific IgE in serum (diluted 1:810) and BALF (undiluted) was measured by rat basophil leukemia (RBL-2H3) cells degranulation assay as described previously (28). The level of total IgA was measured in BALF (diluted 1:20) by ELISA quantification kit (Bethyl, USA) according to the instructions of the manufacturer.

The whole-bacterial cell enzyme-linked immunosorbent assay was used to determine the level of untreated and heat-treated Bl 7952-specific IgA (29). The 96-well microtiter plates were coated by 25 µg of dry mass of bacteria UN or HT in PBS per well and fixed by 0.1% glutaraldehyde for 30 min. Plate was incubated in 0.1% bovine serum albumin (BSA, Sigma Aldrich) with 0.1 M glycine for 2 h. Wells were aspirated, and plate was incubated overnight at 4°C with 0.25% BSA and magnesium chloride (Sigma Aldrich). Samples of BALF (diluted 1:1) were added and after 3-h incubation, the bacteria-specific IgA was detected using horse-radish peroxidase-conjugated IgA antibody (1:10,000; Bethyl, USA) according to the instructions of the manufacturer.

### Statistical Analysis

Data are expressed as mean ± standard deviation (SD). For *in vitro* experiments, the significance of data obtained for UN/HT bacteria treatment was analyzed by unpaired Student’s t-test. Comparison between OVA group and bacteria groups in OVA-sensitized splenocytes was analyzed by one-way ANOVA with Dunnett’s multiple comparison test. In *in vivo* experiment for comparisons between PBS/OVA and bacteria-treated experimental groups, one-way ANOVA with Dunnett’s multiple comparison test was used. A significant difference was considered to exist when the *p-value* was <0.05. Statistical analysis was performed using GraphPad Prism 8.1 Software (San Diego, CA, USA). ∗∗∗∗p < 0.0001, ∗∗∗p < 0.001, ∗∗p < 0.01, ∗p < 0.05; ns, no significance.

## Results

### Heat Treatment Alters Bacterial Surface, Size and Zeta Potential

**Figure 2:**
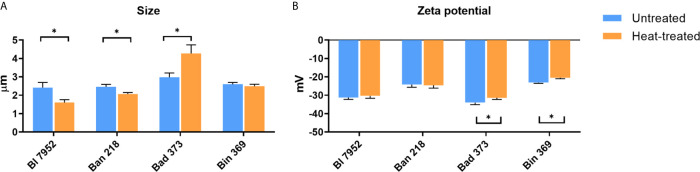
Physicochemical differences between untreated and heat-treated bifidobacteria. **(A)** Size (µm) and **(B)** zeta potential measurements (mV) of untreated (UN, blue bars) and heat-treated (HT, orange bars) bifidobacteria strains were performed using Zetasizer Nano ZS. Data are shown as mean ± SD of five measurements; statistical differences were analyzed by multiple t-tests between UN and HT, *p < 0.05 statistically significant difference.

In order to evaluate the morphological differences in *Bifidobacterium* strains resulting from heat treatment, scanning electron microscopy at low voltage, DLS, and ELS analysis were performed. Heat inactivation of bacteria for 1 h at 65°C induced slight morphological cellular changes (**Supplementary Figure S1**). UN bacteria were characterized by smooth surface in all examined strains, whereas surface of HT bacteria was more porous and irregular. Images of HT bacteria cells revealed no lysis or decomposition of bacterial cells (**Supplementary Figure S1**). DLS analysis showed that the size of UN bifidobacterial strains varies between 2.417 µm (SD ± 0.277) for Bl 7952 strain and 2.979 µm (SD ± 0.234) for Bad 373 strain (**Figure 2A**). After the heat treatment, the bacterial cell size of Bl 7952 and Ban 218 decreased. Contrarily, the cell size of Bad 373 increased and that of Bin 369 remained unchanged. Changes in size did not correspond with changes in bacteria zeta potential (**Figure 2B**) since only two strains, Bin 369 and Bad 373, have significantly lower zeta potential after heat inactivation.

### Heat Treatment Affects Bifidobacteria Engulfment and Cytokine Induction in Epithelial Cells Along With Transfer Capacity Between Epithelial and Dendritic Cells

**Figure 3:**
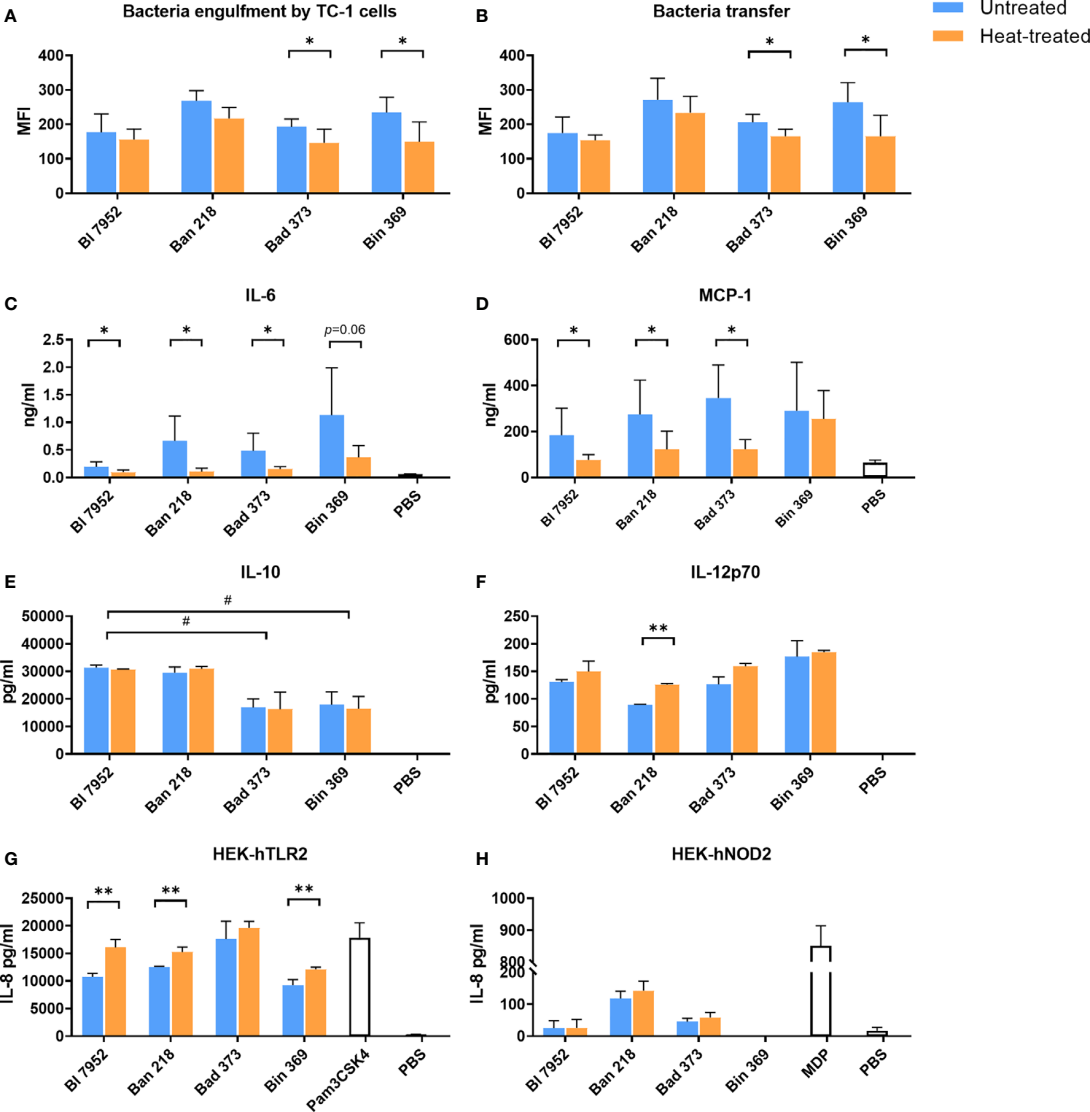
Untreated and heat-treated bifidobacteria strains differently interact with epithelial and immune cells *in vitro*. **(A)** Engulfment by airway epithelial cells and **(B)** transfer between airway epithelial cells and dendritic cells of untreated (UN, blue bars) and heat-treated (HT, orange bars) bifidobacteria. Epithelial cells (TC-1 cells) were incubated overnight with SYTO™ 9-stained bacteria cells. After washing of bacteria, epithelial cells were co-cultured with PKH26-stained JAWS II cells for 4 h. Fluorescence of double stained dendritic cells was analyzed by FACS and expressed as mean fluorescence units (MFI). The level of **(C)** MCP-1 and **(D)** IL-6 cytokine production from TC-1 cells was measured by ELISA in culture supernatants after 24-h stimulation with bifidobacteria (UN or HT) or PBS. The level of **(E)** IL-10 and **(F)** IL-12p70 cytokine production was determined in supernatants of BMDCs after 20-h stimulation with UN or HT bifidobacteria or PBS. Activation of **(G)** TLR2 and **(H)** NOD2 receptors was determined using human embryonic kidney cells (HEK293) stably transfected with human TLR2 or NOD2 expressing vectors and expressed as production of IL-8 cytokine after 20-h stimulation by UN or HT bifidobacteria strains. TLR2 ligand Pam3CSK4 (1 µg/ml) and NOD2 ligand muramyl dipeptide (MDP; 100 ng/ml) were used as positive controls. Cells treated with PBS were used as negative control. Data are collected from three independent experiments. Data are shown as mean ± SD and analyzed with unpaired Student’s t-test between untreated and heat-treated bacteria. *p < 0.05, **p < 0.01 statistically significant difference. Comparison between bifidobacterial strains was calculated by one-way ANOVA Dunett’s multiple comparison test. ^#^p < 0.05.

We assessed whether the observed heat treatment induced changes on bacterial surface properties have an effect on further bacteria processing by epithelial TC-1 cells. We observed a trend towards decreased engulfment of HT bacteria compared to UN bacteria, with a statistically significant decrease for Bin 369 and Bad 373 strains (**Figure 3A**). Next, we focused on bacterial transfer between TC-1 epithelial and antigen presenting cells (JAWS II). In agreement with lower engulfment of HT bacteria, the transfer of these bacteria to the dendritic cells was also lower (**Figure 3B**). In particular, significant decrease was observed for Bin 369 and Bad 373 strains, which are in line with the results of the engulfment experiments (**Figure 3A**). Epithelial and antigen presenting cells were able to produce cytokines and chemokines following the antigen exposure. Upon exposure to UN bifidobacteria, TC-1 cells produced the chemokine MCP-1 and cytokine IL-6 (**Figures 3C, D**); however cytokines such as IL-10, IL-12p70, IFN-*γ*, TGF-*β*, and TNF-α were undetectable (data not shown). Stimulation with HT bacteria was associated with a decrease in both MCP-1 and IL-6 levels compared to UN bifidobacteria. Both UN and HT bacteria triggered cytokine production and maturation of bone-marrow dendritic cells in a species dependent manner (**Figures 3E, F**). The untreated and heat-treated Bl 7952 and Ban 218 strains induced higher levels of IL-10 compared to Bad 373 and Bin 369. On the other hand, stimulation of BMDCs with Bin 369 strain led to the induction of higher levels of IL-12p70 compared to Ban 218. Surprisingly, all bacteria induced the same levels of IL-10 (**Figure 3E**) and IL-12p70 (**Figure 3F**) independently of their viability status. Only in the case of strain Ban 218 that the temperature treatment caused a significant increase of IL-12p70 with no changes in IL-10 levels.

In order to investigate the role of TLR and NOD receptors in bifidobacteria recognition, we examined HEK293 cells stably transfected with hTLR2, hTLR4, hNOD2, and hNOD1 stimulated with both UN and HT bacteria. Bifidobacteria strains were recognized mostly *via* hTLR2 (**Figure 3G**) and only slightly by hNOD2 receptors (**Figure 3H**). No signaling was observed in the case of hTLR4 and hNOD1 (data not shown). The temperature treatment did not change the general recognition of bacteria. In the HEK293-hTLR2 cell line, we observed that in the majority of tested strains, inactivated bacteria showed a stronger receptor activation (**Figure 3G**). In turn, in the HEK293-hNOD2 cell line, no significant differences between UN and HT strains were observed (**Figure 3H**).

### Impact of Bifidobacteria on OVA-Induced Cytokine Production in Splenocyte Cultures From OVA-Sensitized Mice

**Figure 4:**
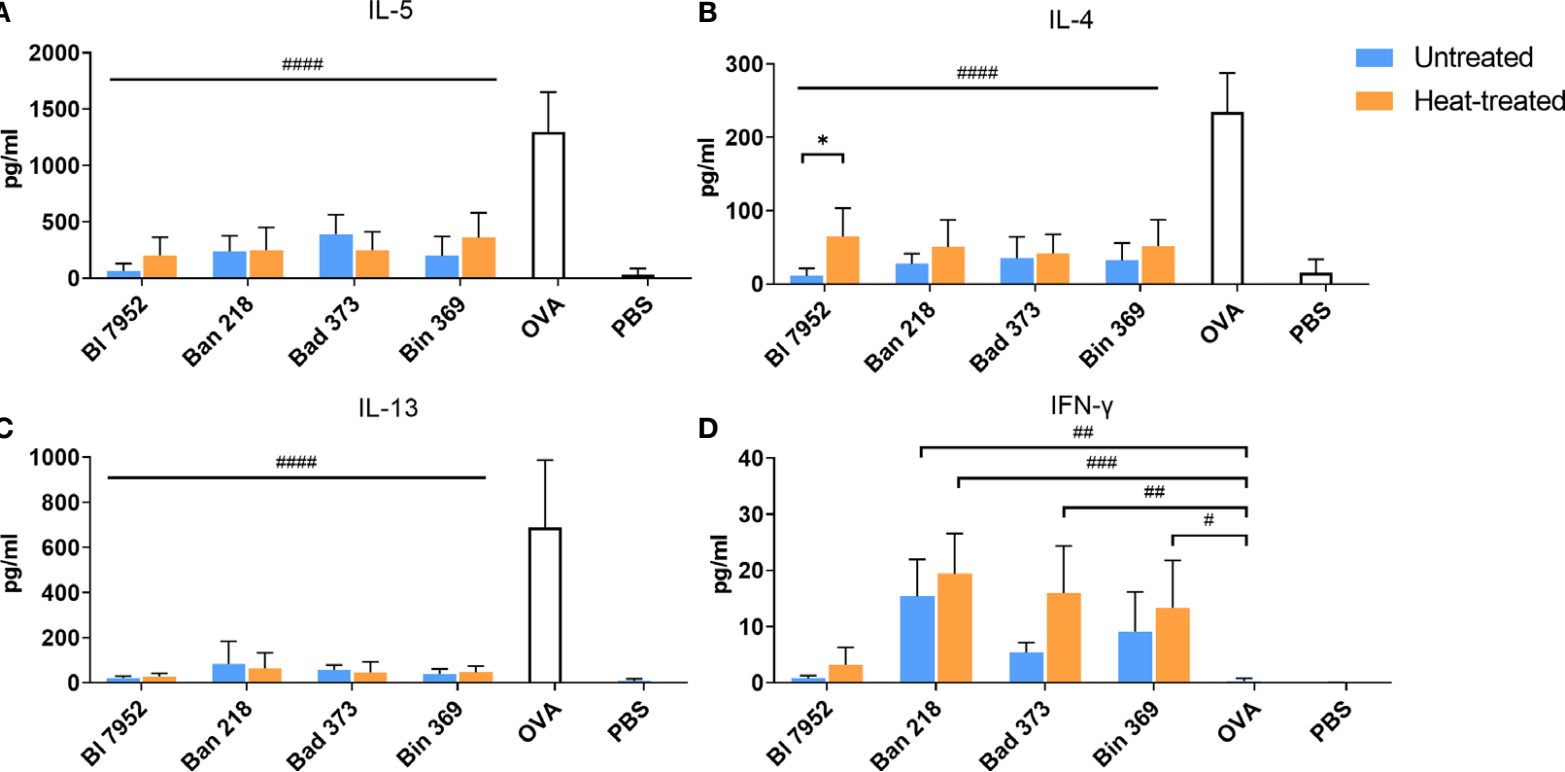
Untreated and heat-treated bifidobacteria strains influence the OVA-induced cytokine production in splenocyte cultures from OVA-sensitized mice. Effect of untreated (UN, blue bars) and heat-treated (HT, orange bars) bifidobacteria strains on cytokine production: **(A)** IL-5, **(B)** IL-4, **(C)** IL-13, and **(D)** IFN-*γ* was measured in supernatants after 72 h cultivation of splenocytes isolated from OVA-sensitized mice and re-stimulated by OVA with or without bifidobacteria strains. Cells stimulated by PBS only served as negative control. Values are pooled from two independent experiments. Significant difference between UN and HT bacteria was estimated by unpaired Student’s t-test (*p <.0.05). Comparison between OVA-stimulated and bifidobacteria + OVA-stimulated groups was calculated by one-way ANOVA Dunett’s multiple comparison test. OVA group was used as control, ^#^p < 0.05, ^##^p < 0.01, ^###^p < 0.001, ^####^p < 0.0001.

We assessed the immunomodulatory potential of UN and HT bifidobacteria on recall OVA-induced cytokines from OVA-sensitized mouse splenocytes. As expected, OVA stimulation induced production of pro-allergic Th2-related cytokines IL-5 (**Figure 4A**), IL-4 (**Figure 4B**), and IL-13 (**Figure 4C**) and no Th1 related IFN-*γ* (**Figure 4D**). Regardless of bacteria viability status, all bifidobacterial strains were able to significantly downregulate the OVA-induced Th2 cytokines. However, in the case of Bl 7952, we observed that HT bacteria caused a weaker IL-4 suppression compared to UN bacteria (**Figure 4B**). Interestingly, the induction of IFN-*γ* was strain dependent (**Figure 4D**). Ban 218 and Bin 369, independently of the viability status, were the most robust inducers of this pro-inflammatory cytokine. On the other hand, Bl 7952 induced negligible level of IFN-*γ* suggesting regulatory, rather than Th1 inducing properties for these bacteria as compared to other tested species.

### Intranasal Administration of Untreated and Heat-Treated Bl 7952 at the Time of Sensitization and Challenge Differently Attenuates the OVA-Induced Cellular and Humoral Immune Responses

In our previous study, Bl 7952 has been shown to prevent allergic sensitization in mono-colonized associated gnotobiotic mice (30). Here we have shown the ability of Bl 7952 to supress OVA-induced Th2 cytokine in conventional mice without inducing Th1 cytokine production in splenocyte cultures from OVA-sensitized mice. It has been suggested that regulatory immune response rather than strongly inducing Th1 response could be more effective in prevention of allergies (31, 32). Therefore, we selected Bl 7952 for further *in vivo* experiments. Bl 7952, either as UN or HT was applied intranasally and impact on the systemic as well as local immune responses was evaluated in mouse model of OVA-induced airway allergy.

**Figure 5:**
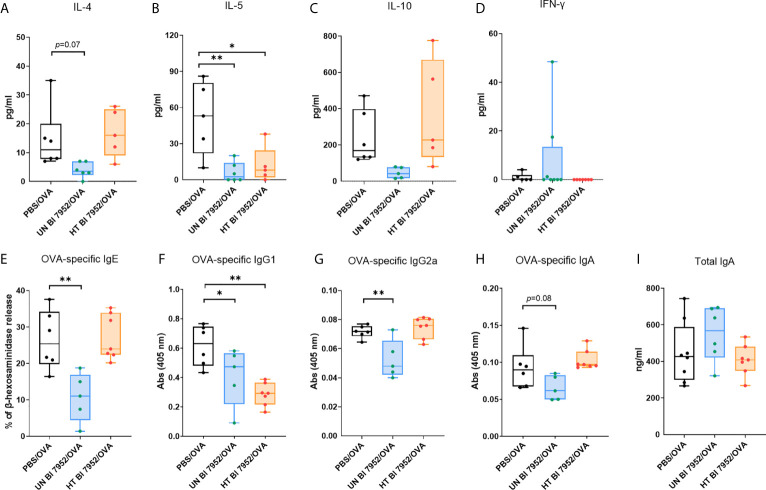
Intranasal administration of untreated and heat-treated Bl 7952 differently attenuates the OVA-induced cellular and humoral immune responses. Effect of PBS (PBS/OVA, black box), untreated Bl 7952 (UN Bl 7952/OVA, blue box) and heat-treated Bl 7952 (HT Bl 7952/OVA, orange box) strain on cytokine production was determined in supernatants of OVA-stimulated splenocytes isolated from bacteria or PBS pre-treated and OVA-sensitized and challenged mice. **(A)** IL-4, **(B)** IL-5, **(C)** IL-10 and **(D)** IFN-*γ* cytokines are expressed as pg/ml. Levels of OVA-specific **(E)** IgE as % of *β*-hexosaminidase release by RBL assay, **(F)** IgG1, **(G)** IgG2a, **(H)** IgA and **(I)** total IgA antibodies were measured in sera of experimental mice. Values are expressed as boxplot with mean and min to max value bars; each dot represents a single mouse. Significant difference between PBS/OVA and bifidobacteria-treated/OVA experimental groups was calculated by one-way ANOVA with Dunnett’s multiple comparison test *p <.0.05. **p < 0.01.

To assess the effect of the Bl 7952 (untreated and heat-treated) treatment on the systemic allergic response of mice we measured the cytokine production from OVA stimulated splenocytes. Administration of untreated Bl 7952 downregulated the Th2-related cytokines IL-4 and IL-5, whereas heat-treated Bl 7952 significantly reduced only the production of IL-5 compared to PBS/OVA group (**Figures 5A, B**). There was no significant difference among the bacteria-treated and PBS/OVA groups in the levels of OVA-induced IL-10 and INF-*γ* cytokines (**Figures 5C, D**). The application of untreated Bl 7952 significantly reduced the serum levels of Th2-related OVA-specific IgE and Th1-related IgG2a antibodies compared to the PBS/OVA group (**Figures 5E, G**). We did not observe this effect after administration of heat-treated Bl 7952. Furthermore, Th2-related OVA-specific IgG1 antibodies (**Figure 5F**) were significantly reduced by both groups of Bl 7952 when compared to PBS/OVA group. There were no differences among the groups in the levels of OVA-specific and total IgA antibody (**Figures 5H, I**).

### Intranasal Administration of Untreated and Heat-Treated Bl 7952 at the Time of Sensitization and Challenge Differently Reduces Allergic Inflammation in Lungs of OVA-Sensitized Mice

**Figure 6:**
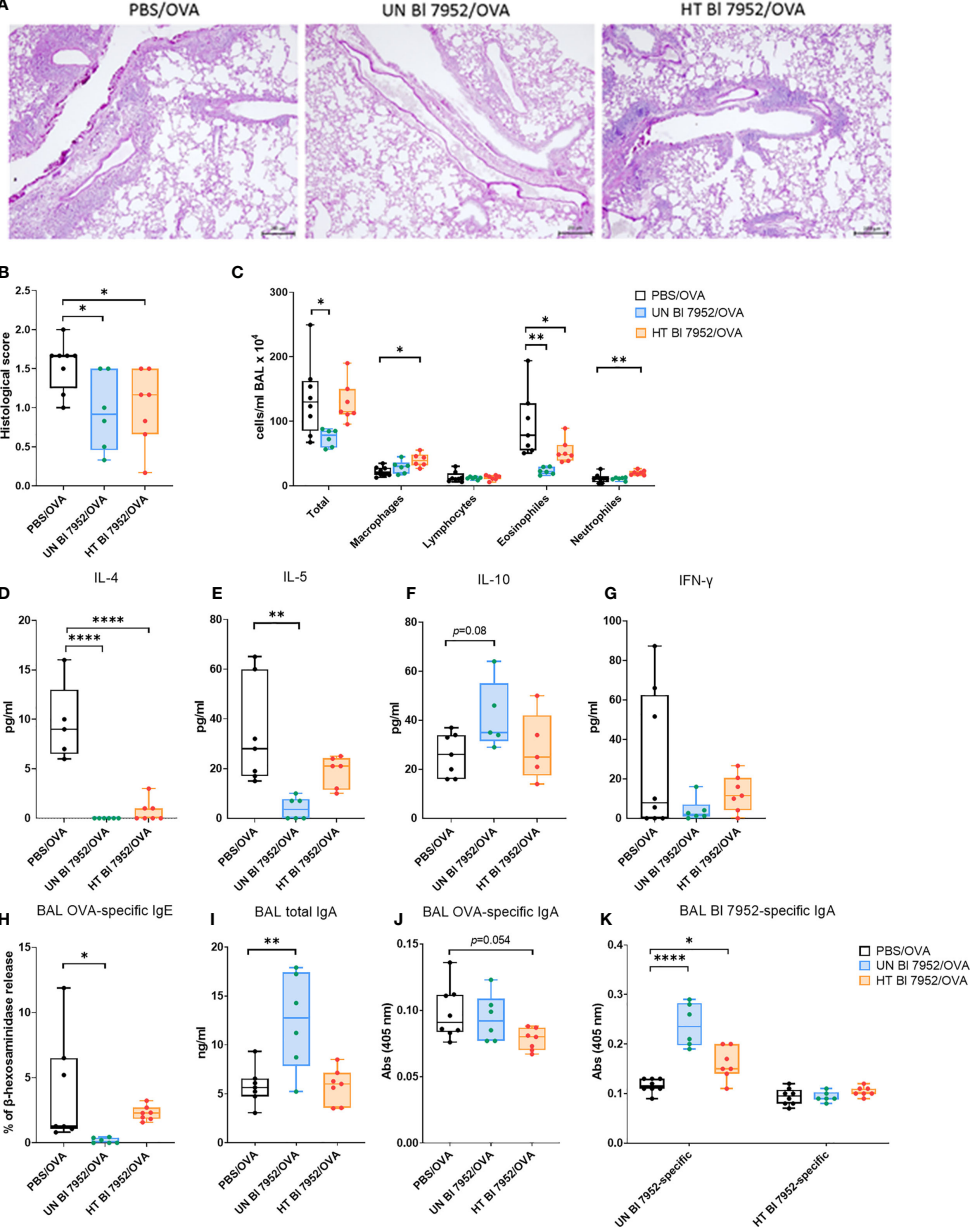
Intranasal administration of untreated and heat-treated Bl 7952 differently reduces allergic inflammation in lungs of OVA-sensitized mice **(A)** Representative tissue section Periodic Acid-Schiff staining of the lungs from OVA-sensitized and challenged mice pre-treated with PBS (PBS/OVA, black box), untreated Bl 7952 (UN Bl 7952/OVA, blue box) and heat-treated Bl 7952 (HT Bl 7952/OVA, orange box) strain. Scale (200 μm) applies to all presented images, magnification ×100. **(B)** Inflammatory score of the lungs was determined by histopathological analysis. **(C)** Total and differential cell counts were determined in bronchoalveolar lavage (BAL). Levels of spontaneous cytokine production from BAL fluid **(D)** IL-4, **(E)** IL-5, **(F)** IL-10 and **(G)** IFN-*γ* were expressed as pg/ml. **(H)** OVA-specific IgE was determined in BAL fluid as % of *β*-hexosaminidase release assay. **(I)** Total IgA in BAL fluid was measured by ELISA and expressed as ng/ml. **(J)** OVA-specific IgA and **(K)** untreated or heat-treated Bl 7952-specific IgA were determined in BAL fluid as optical density (*λ* = 405 nm). Results are shown as boxplot means and min to max value bars; each dot represents a single mouse. Significant difference between PBS/OVA and bifidobacteria-treated/OVA experimental groups was calculated by one-way ANOVA with Dunnett’s multiple comparison test *p <.0.05. **p < 0.01, ****p < 0.0001.

Lung histological analysis showed a reduction of cell infiltration, a lower percentage of mucus-producing cells, decreased perivascular and peribronchiolar inflammation together with lower presence of leukocytes in alveolar spaces in both Bl 7952/OVA groups as compared to control PBS/OVA group (**Figures 6A, B**). The reduced lung allergic inflammation was further confirmed by cellular differential count showing significant decrease in the number of eosinophils in the bronchoalveolar lavage (BAL) of both Bl 7952/OVA groups when compared to the PBS/OVA group. However, the intranasal administration of heat-treated Bl 7952 led to increased amount of neutrophils and macrophages, and no decrease in counts of total cell number in BAL compared to PBS/OVA group (**Figure 6C**). The results of BAL cytokine analysis showed that administration of untreated Bl 7952 significantly decreased the level of IL-4 and IL-5 and increased the level of regulatory cytokine IL-10 compared to the PBS/OVA group (**Figures 6D–F**). The heat-treated Bl 7952 also significantly decreased IL-4, but the levels of IL-5 and IL-10 remained unchanged compared to the PBS/OVA group. We did not observe any changes in the levels of IFN-*γ* among tested groups (**Figure 6G**).

Next, we measured the humoral immune response in the BAL. Results showed that only untreated Bl 7952 was able to reduce the levels of OVA-specific IgE antibodies as shown by IgE-dependant basophil degranulation assay (**Figure 6H**). Moreover, only untreated Bl 7952 significantly increased the levels of total IgA in BAL compared to PBS/OVA (**Figure 6I**). Surprisingly, OVA-specific IgA levels were not significantly different among the groups (**Figure 6J**). This prompted us to assess the bacteria specific IgA levels. When the plate was coated with bacterial mass of untreated Bl 7952, we observed significantly higher levels of specific IgA in BAL of untreated Bl 7952/OVA group compared to PBS/OVA. Heat-treated Bl 7952/OVA group showed higher levels of specific IgA in BAL when compared to OVA/PBS group (**Figure 6K**). Contrary, when the plate was coated with dry mass of heat-treated Bl 7952, level of specific IgA antibodies in BAL did not differ between studied groups and remained low.

## Discussion

In the current study we compared four *Bifidobacterium* strains in order to evaluate the impact of heat inactivation (treatment) on their physical and immunomodulatory properties. We selected the strain *Bifidobacterium longum* ssp. *longum* CCM 7952 (Bl 7952) to investigate *in vivo* the influence of its viability status on the prevention and modulation of the allergic immune response to OVA in a mouse model of allergy.

The classical definition of probiotics states that bacteria must be alive in order to be beneficial for human organism, but recent research suggests that inactivated bacteria are able to maintain the desired beneficial properties (33, 34). The use of inactivated microorganisms expands the possibility of using probiotics in the elderly, neonates, or people with compromised immune system (35, 36). Here, we have shown that mild heat treatment inactivates the bacteria without disruption of the cells, which Taddese et al. considered to be the most important factor in choosing the optimal inactivation method (37). Other inactivation methods such as chemical treatment (38), ultraviolet rays (39), and sonication (40) may be associated with bacterial cell wall damage. Nevertheless, heat treatment induced changes in the structure of the cell wall of all bifidobacterial species tested, resulting in a more irregular and porous appearance compared to untreated bacteria as documented by electron microscopy.

Our subsequent studies of cell size and zeta potential of *Bifidobacterium* show significant physical differences after heat treatment. Similar changes were reported by Baatout at al. who observed that increased temperature leads to an increase in cell size of *E. coli* and, on the contrary, to a decrease in cell size of *Shewanella oneidensis* (41). Such changes may lead to alteration of surface structure and membrane permeability of bacteria. Some proteins might undergo structural changes, denaturation or aggregation, and thus lose their active structure (42). Consequently, important epitopes recognized by the immune system may be hidden or revealed. Finally, the use of high temperatures can lead to the loss of D-alanine in teichoic acids (43), resulting in chelation of magnesium ions in the cell wall, thus altering the surface charge of bacteria.

Interestingly, we have shown that the changes in size of the bacterial strains upon thermal treatment have no significant effect on the degree of their engulfment and transfer into immune cells, in contrast to the changes in zeta potential. The surface electric charge of the two out of the four tested strains dropped significantly after thermal inactivation compared to untreated bacteria. At the same time, we observed marked decrease in their engulfment by epithelial cell line and, as a possible consequence, a decrease in bacterial engulfment by immune cells. Our results are consistent with a study by Galdeano et al. who conducted similar research with live and heat-treated *Lactobacillus* strains. After feeding the bacteria to mice they showed that live bacteria have higher impact on immune cells and live and inactivated bacteria may have different affinity to bind to immune cells (44).

Usually, the probiotic bacteria are administered *via* the oral route. However, recent studies indicate the niche-specific effect of nasal administration of probiotics (10, 11) and the interaction with the immune system and the epithelium of the upper and lower airways. Therefore, we investigated the immunomodulatory properties of tested bacteria on the mouse lung epithelial cell line (TC-1). Interestingly, UN and HT bacteria elicit different levels of IL-6 and MCP-1. These cytokines are involved in macrophage recruitment and activation, which are associated with regulation of the airway inflammation (45). In contrast, bacteria-stimulated dendritic cells showed no changes in maturation and cytokine induction between UN and HT bacteria. It is well established that high IL-12 production by DC, matured by microbial stimuli, leads to Th1 polarization (46) and conversely, high IL-10 production leads to Treg or Tr1 polarization (47). In agreement with Weiss et al., our data showed that analyzed bifidobacteria strains induce low levels of IL-12p70 cytokine and high levels of IL-10 when added to BMDCs. The ability to induce cytokines appears to be specific to each bifidobacterial strain, but is not dependent on viability status, with the exception of strain Ban 218. These results prompted us to ask about differences in the activation of pattern recognition receptors by UN and HT bifidobacterial strains.

We showed that all tested bifidobacterial strains strongly activate the TLR2 receptor. Activation of this receptor is desirable when evaluating bacteria with probiotic properties, as TLR2 activation has been associated with the induction of immunomodulatory and anti-inflammatory effects (48). In addition, it is well documented that TLR2 ligands present on live or killed bacteria in bacterial extracts or surface components, are able to inhibit the allergic response (49, 50). Interestingly, we observed a significant increase in the activation of TLR2 after heat treatment of most tested *Bifidobacterium* strains. This could be due to changes in the structure of the cell wall that facilitate the access of the binding molecule to the receptor (51).

We further focused on the effect of thermal inactivation on the ability of tested *Bifidobacterium* strains to downregulate the cellular recall immune response to allergen in splenocytes from mice sensitized with OVA. Our data show that all strains suppressed the production of the pro-allergic cytokines IL-4, IL-5, and IL-13, but only strain Bl 7952 did not upregulate the pro-inflammatory IFN-*γ*. Next, we observed that although the general profile of immunomodulation was similar between both UN and HT bacteria, significant differences emerged for some strains depending on viability status. In this context, Lopez et al. found that both live and UV-inactivated *Lactobacillus rhamnosus* GG induced the same reduction in the pro-inflammatory cytokine IL-8 upon flagellin induction in Caco-2 cells, but through activation of different signaling pathways (20). Interestingly, inactivation with subsequent loss of viability and cell lysis may induce further and more complex immunomodulation than expected (52).

In our previous studies, we have shown that neonatal mono-colonization with Bl 7952 reduces allergic sensitization, likely through activation of regulatory responses *via* TLR2, MyD88, and MAPK signaling pathways (30). In addition, Bl 7952 administration also protects epithelial barrier integrity during intestinal inflammation (53). Based on these studies and results from *in vitro* experiments we selected Bl 7952 to test its ability to prevent the development of allergic inflammation in a mouse model of airway allergy. Intranasal administration of strain Bl 7952 prior to allergen sensitization and challenge significantly suppressed the allergic immune response in viability status-dependent manner. We showed that intranasal administration of UN bacteria was able to significantly decrease the level of allergen-specific IgE, IgG1, and IgG2a antibodies in sera. On the other hand, heat-treated Bl 7952 markedly reduced only the level of OVA-specific IgG1. Decrease in allergic sensitization should be associated with significant decrease of allergen-specific antibody; however studies have shown that the beneficial effect of probiotic treatment may also occur in the absence of specific antibodies reduction (54).

At the local level, we observed that Bl 7952, depending on viability, significantly decreased the number of pulmonary eosinophils, with or without an increase in neutrophil and macrophage numbers. These results are consistent with the properties of *B. breve* MRx0004, whose therapeutic and prophylactic administration reduced the level of eosinophils and increased the number of macrophages in the mouse asthma model (8). At the same time, we observed a significant reduction in IL-4 and IL-5 in BAL of both Bl 7952 treated groups. IL-5 reduction in BAL fluids was previously shown to correspond with reduced lung inflammation in a mouse model of allergic polysensitization (32). Surprisingly, we observed that UN bacteria have a different ability to induce IL-10 compared to HT bacteria in BAL. IL-10 has a potent immunosuppressive capacity and essentially contributes to allergen tolerance (55).

Along with decreased Th-2 cytokine levels, we observed also a decrease in OVA-specific IgE in BAL both UN and HT Bl 7952-treated groups. This was accompanied by an increase in total IgA in the UN Bl 7952-treated group. Surprisingly, there was little difference in OVA-specific IgA in BAL among the groups. This prompted us to test the UN and HT bacteria-specific IgA antibodies. Interestingly, we observed a significant increase in the level of bacteria-specific IgA antibodies in BAL of mice treated with UN Bl 7952, while we did not see any bacteria-specific IgA production in HT Bl 7952 treated group. These data suggest that the heat-sensitive conformational epitopes of cell wall antigens specific for induction of IgA response were altered. Secretory IgA plays a critical role in tolerance induction and maintenance of mucosal homeostasis in the host by opsonizing and neutralizing pathogens and toxins, resulting in immune exclusion and reducing the antigenic burden on the mucosal immune system. Its protective role has also been widely documented in the management of allergic immune response (9, 56–58). Concomitantly, the decrease in IgA level has been associated with a higher likelihood of developing allergies or asthma (59–61). Nevertheless, not all bacterial strains have the ability to stimulate IgA production. Li et al. showed that only three out of five tested probiotic strains of *L. casei* induced IgA production in mice with house dust mite-induced asthma, indicating that the activity to stimulate IgA production is strain-dependent (9).

**Figure 7:**
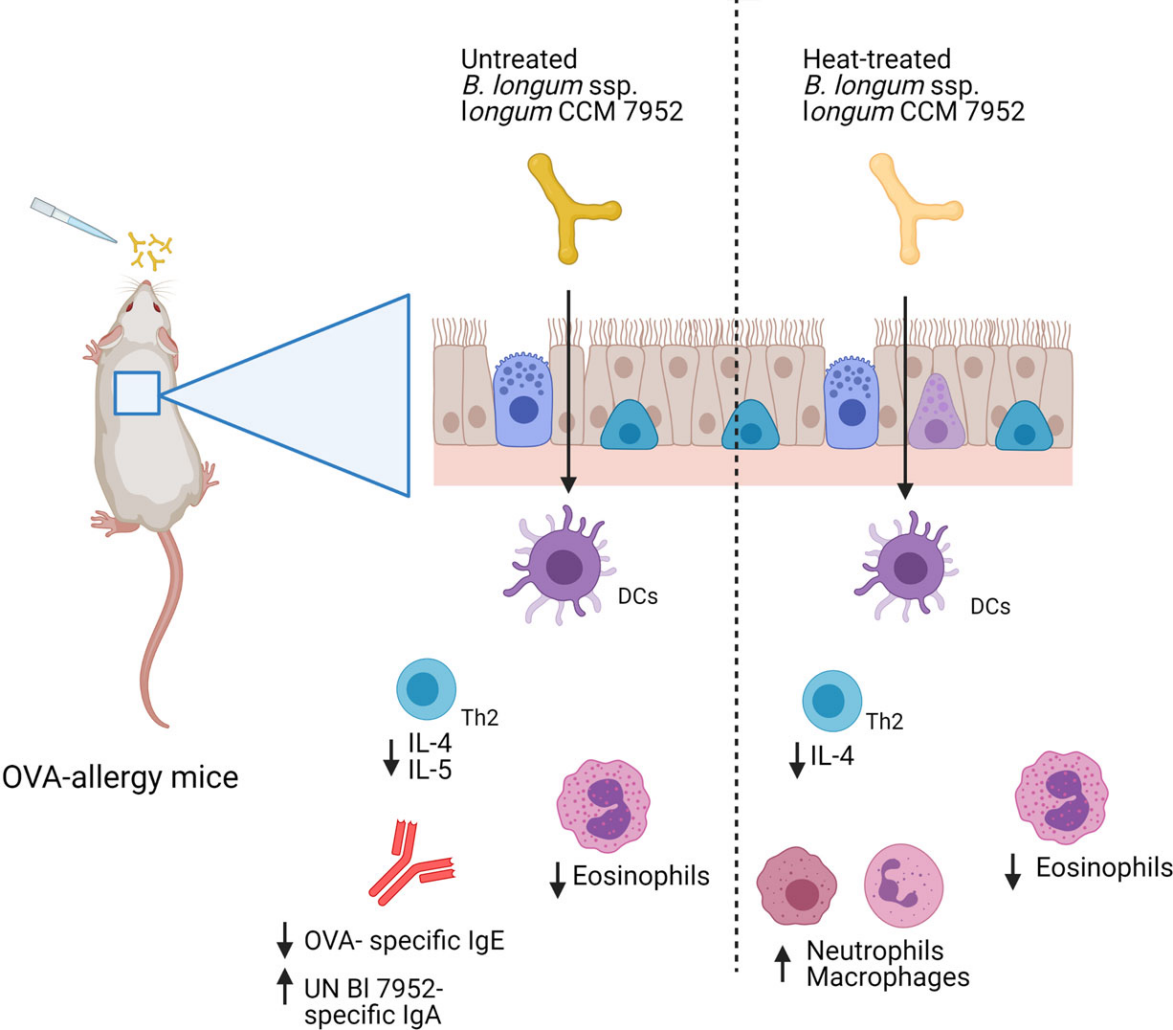
Graphical representation of results. Intranasal administration of Bl 7952 to OVA-sensitized mice suppresses allergic inflammation. However, thermal inactivation of Bl 7952 modifies the immunomodulatory properties of this strain (Created with BioRender.com).

Collectively, we have shown that heat treatment of strain Bl 7952 did result in a decreased ability of this bacterium to prevent and modulate the allergic immune response to ovalbumin in a mouse model of allergy (**Figure 7**). These results were surprising given the observed moderate effect of heat treatment on the immunomodulatory properties of the bacterium in *in vitro* assays. Our results caution against the extrapolating *in vitro* results obtained with inactivated bacteria to the general biological effect of bacteria, and advocated testing the activity of both live and inactivated bacteria in *in vivo* experimental animal models. Further research is warranted to elucidate the immunomodulatory molecules isolated from bacterial strains and their potential use in the prevention of airway allergy diseases.

## Data Availability Statement

The raw data supporting the conclusions of this article will be made available by the authors, without undue reservation.

## Ethics Statement

The animal study was reviewed and approved by Institute of Microbiology, The Czech Academy of Sciences (no. 91/2019).

## Author Contributions

MP prepared the bacteria, performed the *in vitro* experiments, cultivated and stimulated the SPL and TC-1 cell lines, measured the OVA-specific antibody, analyzed the results and drafted the manuscript. MS and DS designed the *in vivo* experiments on mice. DS performed the *in vivo* experiments, histology analysis and BALF cell count, drafted the manuscript. AR performed the DLS and ELS measurement, engulfment and transfer research. PH cultivate the SPL and HEK cell line, KP performed the SPL stimulation, TS performed the RBL test and Bl 7952-specific IgA ELISA. MS coordinate and conceived the study, wrote the manuscript. SG was a supervisor of MP and KP, designed, coordinate and conceived of the study, wrote the manuscript. All authors contributed to the article and approved the submitted version.

## Funding

This work was supported by grant co-founded by the National Science Centre of Poland under grant decision number UMO-2017/26/E/NZ7/01202; by the Polish National Agency for Academic Exchange under grant decision number PPN/BIL/2018/1/00005, by grants 19-02261S and 21-19640M of the Czech Science Foundation and 8JPL19046 of the Ministry of Education, Youth and Sports of the Czech Republic. The group of MS is supported by EMBO Installation Grant.

## Conflict of Interest

The authors declare that the research was conducted in the absence of any commercial or financial relationships that could be construed as a potential conflict of interest.
