# Supplementary material for: Viability Status-Dependent Effect of Bifidobacterium longum ssp. longum CCM 7952 on Prevention of Allergic Inflammation in Mouse Model
Source: Front Immunol. 2021 Jul 20;12:707728. doi: 10.3389/fimmu.2021.707728 (PMC8329652; doi:10.3389/fimmu.2021.707728)
Supplement: Supplementary file 1 [file DataSheet_1.docx]

Supplementary Material

**Viability status-dependent effect of *Bifidobacterium longum* ssp*. longum* CCM 7952 on prevention of allergic inflammation in mouse model**

**Marcelina Joanna Pyclik^1^, Dagmar Srutkova^2^, Agnieszka Razim^1^, Petra Hermanova^2^, Tereza Svabova^2^, Katarzyna Pacyga^1^, Martin Schwarzer^2^ and Sabina Górska^1^**

^1^ Laboratory of Microbiome Immunobiology, Hirszfeld Institute of Immunology and Experimental Therapy, Polish Academy of Sciences, Wroclaw, Poland

^2^ Laboratory of Gnotobiology, Institute of Microbiology, Czech Academy of Sciences, Novy Hradek, Czech Republic

**Supplementary Figure 1. Heat treatment changes surface structure of bifidobacteria.** LV-SEM images of (A) untreated and (B) heat-treated Bl 7952 and (C) untreated and (D) heat-treated Bad 373. Scale (1μm) apply to all presented images.

**
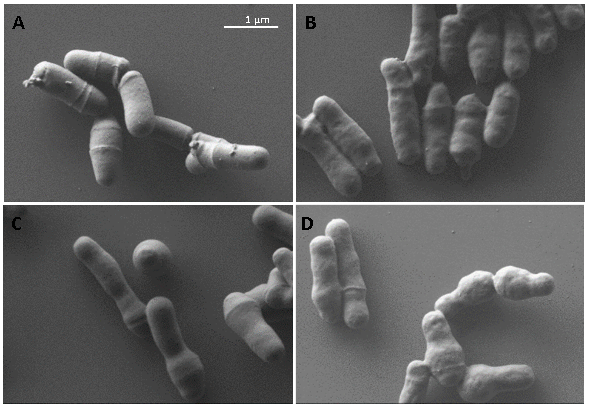
**
